# Diet-Quality Scores and Prevalence of Nonalcoholic Fatty Liver Disease: A Population Study Using Proton-Magnetic Resonance Spectroscopy

**DOI:** 10.1371/journal.pone.0139310

**Published:** 2015-09-29

**Authors:** Ruth Chan, Vincent Wai-Sun Wong, Winnie Chiu-Wing Chu, Grace Lai-Hung Wong, Liz Sin Li, Jason Leung, Angel Mei-Ling Chim, David Ka-Wai Yeung, Mandy Man-Mei Sea, Jean Woo, Francis Ka-Leung Chan, Henry Lik-Yuen Chan

**Affiliations:** 1 Department of Medicine and Therapeutics, The Chinese University of Hong Kong, Hong Kong; 2 Centre for Nutritional Studies, The Chinese University of Hong Kong, Hong Kong; 3 Institute of Digestive Disease, The Chinese University of Hong Kong, Hong Kong; 4 Department of Imaging and Interventional Radiology, The Chinese University of Hong Kong, Hong Kong; 5 Jockey Club Centre for Osteoporosis Care and Control, The Chinese University of Hong Kong, Hong Kong; 6 Department of Clinical Oncology, The Chinese University of Hong Kong, Hong Kong; Medical University Innsbruck, AUSTRIA

## Abstract

Dietary pattern analysis is an alternative approach to examine the association between diet and nonalcoholic fatty liver disease (NAFLD). This study examined the association of two diet-quality scores, namely Diet Quality Index-International (DQI-I) and Mediterranean Diet Score (MDS) with NAFLD prevalence. Apparently healthy Chinese adults (332 male, 465 female) aged 18 years or above were recruited through a population screening between 2008 and 2010 in a cross-sectional population-based study in Hong Kong. DQI-I and MDS, as well as major food group and nutrient intakes were calculated based on dietary data from a food frequency questionnaire. NAFLD was defined as intrahepatic triglyceride content at ≥5% by proton-magnetic resonance spectroscopy. Multivariate logistic regression models were used to examine the association between each diet-quality score or dietary component and prevalent NAFLD with adjustment for potential lifestyle, metabolic and genetic factors. A total of 220 subjects (27.6%) were diagnosed with NAFLD. DQI-I but not MDS was associated with the prevalence of NAFLD. A 10-unit decrease in DQI-I was associated with 24% increase in the likelihood of having NAFLD in the age and sex adjusted model (95% CI: 1.06–1.45, *p* = 0.009), and the association remained significant when the model was further adjusted for other lifestyle factors, metabolic and genetic factors [OR: 1.26 (95% CI: 1.03–1.54), *p* = 0.027]. Multivariate regression analyses showed an inverse association of the intake of vegetables and legumes, fruits and dried fruits, as well as vitamin C with the NAFLD prevalence (*p*<0.05). In conclusion, a better diet quality as characterized by a higher DQI-I and a higher consumption of vegetables, legumes and fruits was associated with a reduced likelihood of having NAFLD in Hong Kong Chinese.

## Introduction

Nonalcoholic fatty liver disease (NAFLD) is the most common cause of abnormal liver biochemistry worldwide. Its prevalence is increasing in Asia [1–3]. The worldwide prevalence of NAFLD in general population is estimated to be 20 to 30% in Western countries and 5 to 18% in Asia [3]. NAFLD may progress to a wide range of liver abnormalities, ranging from nonalcoholic steatohepatitis (NASH), cirrhosis, liver failure and liver cancer. It is also considered as the leading etiology for cryptogenic cirrhosis [4,5]. Substantial evidence supports that NAFLD, obesity and metabolic syndrome are strongly correlated, and NAFLD is an independent cardiovascular risk factor [6,7]. Therefore, determining risk factors for hepatic steatosis are important.

Diet may affect the development of NAFLD, and dietary alterations have been suggested as a cornerstone for the management of NAFLD [8]. However, epidemiological data on the association between diet or nutrient composition and the development of NAFLD are limited. Previous studies on this aspect were mainly conducted in Caucasian populations. Most previous studies were limited by small sample size [9,10] and focused on high-risk population or NAFLD patients [11–14]. Only few studies have included participants from a representative general population [15–17]. Moreover, the examination of diet as a risk factor for NAFLD has traditionally focused on the effect of single foods and nutrients, such as soft drinks and meat [16], fructose [18], choline [19] and n-3 polyunsaturated fatty acids [17]. Recently, a possible link between dietary acid-base load and NAFLD has also been suggested [20,21]. However, since diet is a combination of multiple exposures of food and nutrients, dietary pattern analysis is considered to be an alternative approach to investigate the association between diet and disease risk in epidemiological studies [22]. This approach involves the examination of the whole diet and considers the synergy of food and nutrient consumption, thus it may be more predictive of disease risk in comparison to individual foods or nutrients [22,23].

To our knowledge, only few observational studies [24–29] have been conducted to examine the association between dietary patterns and NAFLD in adults and one prospective study has been done in a population-based cohort of adolescents [30]. In view of the scarcity of evidence on this topic and the fact that Chinese diets are different from Western diets, the present study aimed to examine the association of two diet-quality scores, namely the Mediterranean Diet Score (MDS) and the Diet Quality Index-International (DQI-I) with NAFLD prevalence in 797 Chinese aged 18 years or above, using data from a population-based study in Hong Kong. The MDS was selected because the Mediterranean diet has been widely reported for its many health benefits including its potential protective effect against NAFLD [31,32]. The traditional Chinese diet also shares several similarities with the Mediterranean diet, such that the MDS can be applied to Chinese population [33]. The DQI-I was chosen as it provides an effective means of cross-national comparative work for global understanding of diet quality, and has been used in epidemiological studies to quantify the diet quality in Chinese population [34,35]. We hypothesized that higher diet-quality score was associated with a reduced likelihood of NAFLD.

The present analysis was different from the findings regarding the possible link between dietary acid-base load and NAFLD of the same study population [20] in regard to the approach of analyzing dietary data. The present analysis used the dietary pattern approach in which the synergy of food and nutrient consumption was considered, while the previous analysis considered the association of a single nutritional factor, namely dietary acidity with the likelihood of NAFLD. Otherwise, both analyses followed the same study protocol (S1 and S2 Files).

## Materials and Methods

### Study population

The study population were 797 subjects who aged 18 years or above and participated in a cross-sectional population screening study for NAFLD. Details of the screening project have been reported previously [36,37] (S1 File) and can be found in the study protocol (S2 File). Those with active malignancy, metallic implants or other contraindications to magnetic resonance imaging, positive hepatitis B surface antigen or antibody against hepatitis C virus, treatment with steatogenic drugs (e.g. corticosteroids and estrogens), secondary causes of fatty liver (e.g. consumption of amiodarone and tamoxifen) and decompensated liver disease (defined as bilirubin above 50 μmol/l, albumin below 35 g/l, platelet count below 150 × 10^9^/l, international normalized ratio above 1.3, or the presence of ascites or varices) (n = 147) were excluded [36,37]. The present study also excluded those with excessive alcohol use (defined as more than 20 g/d day in men and more than 10 g/d in women) (n = 30) and those with incomplete dietary data (n = 95) from the final analysis. The study protocol conformed to the ethical guidelines of the Declaration of Helsinki, and was approved by the Clinical Research Ethics Committee of The Chinese University of Hong Kong. All subjects provided informed written consent.

### Clinical assessment

At the baseline clinic visit, information of drug history, alcohol intake, smoking and past medical history were collected using a standardized questionnaire. Anthropometric measurements including body weight, body height, waist and hip circumferences were obtained using standardized methods. Blood tests for liver biochemistry, glucose and lipids were taken after at least 8 hours of fasting. Metabolic syndrome was defined according to the ethnic-specific criteria by the International Diabetes Federation, which was modified from the National Cholesterol Education Program, Adult Treatment Panel III Guidelines [38]. Details of the clinical assessment have been described previously [36].

### Proton-magnetic resonance spectroscopy (^1^H-MRS)

^1^H-MRS was performed to measure intrahepatic triglyceride content (IHTG) within 8 weeks from the baseline visit. Details of the scanning sequence and analysis have been described previously [36,39]. Whole-body 3.0T scanner with a single voxel point-resolved spectroscopy sequence and an echo time of 40 ms and repetition time of 5,000 ms was used. Fatty liver was defined as an IHTG of 5% or more [40].

### Genetic analysis

Genomic DNA was extracted from 100 μl buffy coat using QIAamp Blood DNA Mini Kit (Qiagen, Germany) and QIAcube System (Qiagen, Germany). Extracted DNA was quantified using Nanodrop 1000 (Thermo Fisher Scientific, USA). For each subject, 20 ng of genomic DNA was used for *PNPLA3* rs738409 allelic discrimination using TaqMan® SNP Genotyping Assays (Life Technologies, USA) on the Applied Biosystems 7900HT Fast Real-Time PCR System (Life Technologies, USA). Details have been described elsewhere [41].

### Dietary assessment

A locally validated food-frequency questionnaire (FFQ) was used to capture food intake and dietary intake over 7 days [42]. The questionnaire contained 297 food items in seven broad categories: bread / pasta / rice; vegetables; fruit; meat / fish / egg; beverages; dimsum / snacks; soups; and oil / salt / sauces. Subjects were asked to complete the questionnaires under the supervision of a trained research staff, with food models, food containers, and a catalogue of pictures of individual food portions provided to facilitate portion size estimation. The amount of cooking oil was calculated based on the usual cooking methods, the usual type of cooking oil and the usual portion of different foods used by the subjects [43]. Daily nutrient intakes and food group intakes were estimated using Food Processor Nutrition analysis and Fitness software version 8.0 (ESHA Research, Salem, Oregon, USA) with the addition of local and Chinese food items [44–46]. All nutrient intakes were energy adjusted by the residual method for regression analysis [47]. Details of the dietary assessment have been described previously [20].

### The Mediterranean Diet Score (MDS)

Adherence to the Mediterranean diet was calculated using the revised method described by Trichopoulou *et al*. [48]. One score was assigned to consumption of the food groups considered beneficial to health at or above the sex-specific median (vegetables, legumes, fruits and nuts, cereal, fish and monosaturated to saturated lipids ratio). Similarly, one score was also assigned to consumption of the food groups presumed to be detrimental to health (meat, poultry and dairy products) below the median. Because a substantial proportion of subjects did not consume alcohol, the component of ethanol consumption was excluded in the scoring. Therefore, total MDS ranged from 0 (minimal adherence) to 8 (maximal adherence) instead of 0 to 9.

### Dietary Quality Index-International (DQI-I)

The Dietary Quality index-International (DQI-I) was calculated according to the method described by Kim *et al*. [34]. Four major aspects of the diet are assessed in the DQI-I, namely variety, adequacy, moderation and overall balance. The score ranges from 0 to 100 and higher score represents better diet quality. In this study, we did not have enough information to calculate the category of empty-calorie foods under the aspect ‘moderation’. Therefore the range of score for moderation was 0 to 24 instead of 0 to 30 as originally proposed in the calculation, and the DQI-I total score was 0 to 94 instead of 0 to 100. Details of the DQI-I calculation have been described elsewhere [49].

### Statistical analysis

Statistical tests were performed using the Statistical Package for Social Sciences version 21.0 (SPSS Inc., Chicago, US). Histograms were used to screen for normal distribution. Logarithmic transformation was applied to skewed variables whenever appropriate. Continuous variables were expressed in mean ± SD and compared using the independent *t* test if they were normally distributed. Skewed variables were expressed in median (IQR) and compared using the Mann-Whitney U test. Categorical variables were compared using chi-squared test or Fisher exact test as appropriate. Hardy–Weinberg equilibrium of alleles was assessed by chi-squared test. Pearson’s correlation or Spearman’s rank correlation was used to examine the correlation between each diet-quality score and food group or nutrient intakes whenever appropriate.

The association between each diet-quality score and IHTG and the presence of NAFLD was analyzed using multivariate linear regression and logistic regression models, respectively. The first model was adjusted for sex and age (continuous). The second model was further adjusted for BMI (continuous), daily energy intake (continuous), current smoker status (yes/no), current drinker status (yes/no), the five individual metabolic components including central obesity (yes/no), triglyceride >1.7 mmol/l (yes/no), reduced HDLC (yes/no), hypertension (yes/no), and impaired fasting glucose or diabetes (yes/no), and the *PNPLA3* genotypes. We also examined whether the association between each diet-quality score and the presence of NAFLD varied according to sex, BMI, age, current drinker status, the presence of metabolic syndrome, or the *PNPLA3* genotypes. All models were additionally stratified by sex (men and women), age (<50 vs. > = 50 years), BMI (normal weight group <23 vs. overweight and obese group > = 23 kg/m^2^), current drinker status (yes vs. no), the presence of metabolic syndrome (yes vs. no), and the *PNPLA3* genotypes (CC vs. CG vs. GG genotypes). We also investigated potential effect modifications by sex, age, BMI, current drinker status, the presence of metabolic syndrome and the *PNPLA3* genotypes by inclusion of cross-product terms to the regression models. No significant interactions were detected for all these stratified variables, thus data were presented based on the results of the whole sample. The above multivariate logistic regression models were repeated to explore the association between each energy adjusted nutrient intake variable or food group intake variable and the prevalence of NAFLD. Due to the skewness in the distribution for most food group intake variables, the food group intake variables were categorized into tertiles based on the distribution of the entire sample. Odds ratios (OR) were computed to compare the middle and upper tertile groups with the lower tertile group. Test for trend was examined by entering tertiles of food group intake as a fixed factor and testing the contrast by using the polynomial option in all models. All tests were 2-sided and *P* values less than 0.05 were considered statistically significant.

## Results

There was no major difference in the baseline characteristics between the included subjects and the excluded subjects (data not shown). The mean ± SD age of 797 subjects was 48.1 ± 10.6 years (range 19–72 years), and 332 (41.7%) were male. All subjects were ethnic Chinese. Two hundred and twenty subjects were diagnosed with NAFLD and the prevalence of NAFLD was 27.6%. Subjects with NAFLD were older, and were likely to be male and current drinkers than subjects without NAFLD (all *p*<0.05). They also had significantly higher BMI and higher WC than those without NAFLD. The metabolic profiles differed significantly between the two groups. Subjects with NAFLD showed lower age and sex adjusted MDS and DQI-I than subjects without NAFLD (Table 1). There were 311 (39.0%) CC homozygotes, 380 (47.7%) CG heterozygotes and 105 (13.2%) GG homozygotes. The alleles of *PNPLA3* rs738409 polymorphism of the total study sample or of the stratified sample by NAFLD status were in Hardy-Weinberg equilibrium (*p* = 0.511 for total sample, *p* = 0.533 for non-NAFLD group, *p* = 0.414 for NAFLD group).

**Table 1:** Baseline subject characteristics between subjects with and without NAFLD (n = 797).

	No NAFLD (n = 577)	NAFLD (n = 220)	*P* ^1^	Age and sex adjusted *P* ^2^
Age (years)	47.0 ± 10.8^3^	51.0 + 9.3	<0.001	—-
BMI (kg/m^2^)	21.7 ± 3.0	25.5 + 3.5	<0.001	<0.001
WC (cm)	78.4 ± 9.2	89.2 + 7.6	<0.001	<0.001
Male [n (%)]	214 (37.1)	118 (53.6)	<0.001	—-
Current smoker [n (%)]	48 (8.3)	19 (8.6)	0.885	0.504
Current drinker [n (%)]	96 (16.6)	55 (25.0)	0.007	0.177
MDS	4.0 ± 1.7^3^	4.0 + 1.8	0.904	0.068
DQI-I	57.9 ± 10.4	57.2 + 11.5	0.368	0.007
SBP (mmHg)	126.1 ± 19.0	137.8 + 19.0	<0.001	<0.001
DBP (mmHg)	79.2 ± 11.3	88.3 + 12.6	<0.001	<0.001
Alanine aminotransferase (IU/l)	19 (15–25)	31 (23–42)	<0.001^4^	<0.001
Fasting glucose (mmol/l)	4.9 (4.6–5.2)	5.2 (4.9–5.6)	<0.001^4^	<0.001
Total cholesterol (mmol/l)	5.1 + 1.0	5.4 + 1.0	<0.001	0.012
HDL-cholesterol (mmol/l)	1.6 + 0.4	1.3 + 0.3	<0.001	<0.001
Triglycerides (mmol/l)	0.9 (0.7–1.2)	1.6 (1.2–2.3)	<0.001^4^	<0.001
LDL-cholesterol (mmol/l)	2.9 ± 0.8	3.2 + 0.9	<0.001	0.002
Central obesity^5^ [n (%)]	160 (27.7)	143 (65.0)	<0.001	<0.001
Triglycerides > 1.7 mmol/l^5^ [n (%)]	63 (10.9)	97 (44.1)	<0.001	<0.001
Reduced HDL-cholesterol^5^ [n (%)]	45 (7.8)	64 (29.1)	<0.001	<0.001
Hypertension^5^ [n (%)]	251 (43.5)	161 (73.2)	<0.001	<0.001
Impaired fasting glucose or DM^5^ [n (%)]	53 (9.2)	61 (27.7)	<0.001	<0.001
Metabolic syndrome^5^ [n (%)]	45 (7.8)	105 (47.7)	<0.001	<0.001
*PNPLA3* genotypes [n (%)]				
CC	248 (43.1)	63 (28.6)	<0.001	<0.001
CG	265 (46.0)	115 (52.3)		
GG	63 (10.9)	42 (19.1)		
Intrahepatic triglyceride content (%)	1.2 (0.7–2.4)	9.8 (7.0–15.4)	<0.001^4^	<0.001

^1^ Group difference by independent *t* test for continuous variables and chi-squared or Fisher exact test for categorical variables unless otherwise stated

^2^ Group difference by ANCOVA for continuous variables and logistic regression for categorical variables unless otherwise stated

^3^ Mean ± SD or median (interquarter range) (all such values) unless otherwise stated

^4^ Group difference by nonparametric Mann-Whiney U test

^5^ International Diabetes Federation criteria for metabolic syndrome [38]

BMI, body mass index; DBP, diastolic blood pressure; DM, diabetes mellitus; DQI-I, Diet Quality Index-International; MDS, Mediterranean Diet Score; NAFLD, nonalcoholic fatty liver disease; SBP, systolic blood pressure; WC, waist circumference

Higher MDS and DQI-I was associated with lower intakes of beverages, dim sum, egg and egg products, fast food, meat, poultry and organ meat. Both scores were positively associated with the intakes of fish and seafood, grains and cereals, and plant-based foods, such as fruits, soy and soy products, and vegetables and legumes (Table 2). MDS and DQI-I were positively associated with percentage of energy from carbohydrate and intakes of dietary fiber and vitamin C, and negatively associated with percentage of energy from total fat and saturated fat, and cholesterol intake (all *p*<0.05, Table 2).

**Table 2:** Correlation between each diet-quality score and selected food group and nutrient intakes (n = 797).

Daily food group/nutrient intake^1^	MDS	*P* value	DQI-I	*P* value
Beverages (ml)^2^	-0.157	<0.001	-0.198	<0.001
Dim sum (g)	-0.015	0.671	-0.033	0.353
Eggs (g)	-0.097	0.006	-0.207	<0.001
Fast food (g)	-0.219	<0.001	-0.185	<0.001
Meat, poultry and organ meats (g)	-0.242	<0.001	-0.281	<0.001
Fish and seafood (g)	0.394	<0.001	0.151	<0.001
Fruits (g)	0.508	<0.001	0.604	<0.001
Grains and cereals (g)	0.344	<0.001	0.379	<0.001
Mushroom, fungi and nuts (g)	0.231	<0.001	0.177	<0.001
Soy and soy products (g)	0.350	<0.001	0.235	<0.001
Vegetables and legumes (g)	0.512	<0.001	0.539	<0.001
Milk and milk products (g)	-0.301	<0.001	0.009	0.800
Tea and coffee (ml)	0.030	0.397	-0.016	0.642
% energy from carbohydrate	0.321	<0.001	0.651	<0.001
% energy from protein	0.024	0.505	-0.022	0.539
% energy from total fat	-0.311	<0.001	-0.664	<0.001
% energy from saturated fat	-0.473	<0.001	-0.634	<0.001
Cholesterol (mg)	-0.045	0.201	-0.220	<0.001
Dietary fiber (g)	0.544	<0.001	0.642	<0.001
Vitamin C (mg)	0.502	<0.001	0.615	<0.001

^1^ Spearman’s rank correlation was used for food group intake analysis and vitamin C intake analysis whereas Pearson’s correlation was used for other nutrient intakes analysis

^2^ Included mainly sweetened beverages or juice, carbonated drinks and sports drinks

DQI-I, Diet Quality Index-International; MDS, Mediterranean Diet Score

The multivariate linear regression models suggested that a higher MDS or DQI-I was associated with a lower IHTG (Table 3). Using an IHTG of 5% or more as a cut-off value to define NAFLD, the DQI-I was associated with the prevalence of NAFLD (Table 4). A 10-unit decrease in DQI-I was associated with 24% increase in the likelihood of having NAFLD in the age and sex adjusted model (95% CI: 1.06–1.45, *p* = 0.009), and the association remained significant when the model was further adjusted for other lifestyle factors, metabolic components and the *PNPLA3* genotypes [OR: 1.26 (95% CI: 1.03–1.54), *p* = 0.027]. The multivariate adjusted association of DQI-I and the prevalence of NAFLD was stronger in male than in female, and in overweight or obese subjects than in normal weight subjects. Similar results were observed between the MDS and the prevalence of NAFLD, although the strength of the associations were not significant in comparison to those of the DQI-I with the NAFLD prevalence (Table 4). Since significant correlations were observed between both diet-quality scores and most food groups or nutrients, the association of each major food group or nutrient with the likelihood of having NAFLD was further examined as to get a more concrete picture of the optimal diet in reducing NAFLD. Among all selected food groups and nutrients examined in the multivariate adjusted models, higher intake of vegetables and legumes, fruits and dried fruits, as well as vitamin C was consistently associated with reduced likelihood of having NAFLD (Table 5).

**Table 3:** Linear regression analysis linking each diet-quality score and the intrahepatic triglyceride content^1^ (n = 797).

Diet-quality score	Age and sex adjusted			Multivariate adjusted^2^		
	Beta	SE	*P* value	Beta	SE	*P* value
MDS	-0.036	0.011	0.001	-0.033	0.009	<0.001
DQI-I	-0.006	0.002	0.001	-0.004	0.001	0.003

^1^ Logarithmic transformation was applied for regression analysis

^2^ Further adjusted for BMI, energy intake, current smoker status (yes/no), current drinker status (yes/no), central obesity (yes/no), triglyceride >1.7 mmol/l (yes/no), reduced HDL-cholesterol (yes/no), hypertension (yes/no), impaired fasting glucose or diabetes (yes/no), and the *PNPLA3* genotypes (CC vs. CG vs. GG genotypes)

DQI-I, Diet Quality Index-International; MDS, Mediterranean Diet Score

**Table 4:** Overall and stratified logistic regression analysis linking each diet-quality score and the presence of NAFLD (n = 797).

	No. of	MDS (per 1 unit decrease)						DQI-I (per 10 unit decrease)					
	NAFLD	Age and sex adjusted^1^			Multivariate adjusted^2^			Age and sex adjusted^1^			Multivariate adjusted^2^		
	case/control	OR	95% CI	*P* value	OR	95% CI	*P* value	OR	95% CI	*P* value	OR	95% CI	*P* value
Overall analysis	220/577	1.10	0.99–1.21	0.073	1.11	0.98–1.26	0.092	1.24	1.06–1.45	**0.009**	1.26	1.03–1.54	**0.027**
Stratified analysis													
Sex													
Male	118/214	1.09	0.95–1.25	0.244	1.17	0.98–1.38	0.081	1.30	1.06–1.61	**0.014**	1.34	1.03–1.75	**0.030**
Female	102/363	1.12	0.97–1.29	0.111	1.01	0.85–1.21	0.901	1.20	0.95–1.52	0.134	1.09	0.80–1.47	0.588
Age													
<50 years	92/317	1.07	0.92–1.24	0.401	1.10	0.90–1.33	0.363	1.22	0.97–1.55	0.091	1.23	0.90–1.68	0.202
> = 50 years	128/260	1.04	0.92–1.18	0.497	1.05	0.90–1.23	0.525	1.15	0.94–1.41	0.188	1.11	0.86–1.44	0.410
BMI status													
<23 kg/m^2^	53/426	1.00	0.83–1.21	0.975	1.01	0.81–1.28	0.903	1.05	0.79–1.39	0.739	0.88	0.62–1.25	0.480
> = 23 kg/m^2^	167/147	1.16	1.01–1.33	**0.031**	1.19	1.03–1.38	**0.021**	1.29	1.04–1.61	**0.022**	1.39	1.09–1.77	**0.008**
Current drinker													
Yes	55/96	1.20	0.97–1.48	0.089	1.28	0.97–1.69	0.086	1.14	0.84–1.56	0.394	1.31	0.85–2.03	0.219
No	165/481	1.06	0.95–1.19	0.313	1.05	0.91–1.20	0.511	1.25	1.04–1.51	**0.017**	1.17	0.93–1.47	0.174
Metabolic syndrome													
Yes	105/45	1.09	0.88–1.34	0.437	1.12	0.88–1.42	0.351	1.11	0.81–1.53	0.524	1.22	0.84–1.78	0.288
No	115/532	1.06	0.94–1.21	0.345	1.06	0.91–1.22	0.467	1.22	0.99–1.50	0.059	1.20	0.94–1.53	0.142
*PNPLA3* genotypes													
CC	63/248	1.23	1.02–1.49	**0.029**	1.17	0.93–1.47	0.192	1.31	0.98–1.76	0.073	1.30	0.90–1.88	0.156
CG	115/265	1.06	0.92–1.22	0.445	1.07	0.89–1.28	0.469	1.27	1.02–1.59	0.031	1.22	0.92–1.62	0.176
GG	42/63	1.08	0.87–1.34	0.479	1.16	0.86–1.57	0.330	1.15	0.78–1.68	0.482	1.16	0.67–2.02	0.600

^1^ Adjusted for age and sex, expect for the stratified variable whenever appropriate.

^2^ Further adjusted for BMI, energy intake, current smoker status (yes/no), current drinker status (yes/no), central obesity (yes/no), triglyceride >1.7 mmol/l (yes/no), reduced HDL-cholesterol (yes/no), hypertension (yes/no), impaired fasting glucose or diabetes (yes/no), and the *PNPLA3* genotypes (CC vs. CG vs. GG genotypes), except for the stratified variable whenever appropriate.

DQI-I, Diet Quality Index-International; MDS, Mediterranean Diet Score; NAFLD, non-alcoholic fatty liver disease

**Table 5:** Logistic regression analysis linking individual food groups or nutrients and the presence of NAFLD (n = 797).

Food group/nutrient	Tertile^1^	No. of	Age and sex adjusted			Multivariate adjusted^2^		
		NAFLD	OR	95% CI	*P*_*trend*_ *or*	OR	95% CI	*P*_*trend*_ *or*
		case/control			*P* value^3^			*P* value^3^
Beverages (ml)^4^	0	231/87	1		**0.008**	1		0.091
	1–71	163/47	1.01	0.66–1.54		1.22	0.72–2.08	
	71+	183/86	1.68	1.14–2.47		1.53	0.93–2.52	
Dim sum (g)	<14	202/76	1		0.551	1		0.548
	14–40	188/67	1.07	0.72–1.59		1.06	0.64–1.75	
	40+	187/77	1.13	0.76–1.65		1.16	0.71–1.89	
Egg and egg products (g)	<7	209/79	1		0.754	1		0.471
	7–21	150/57	1.00	0.66–1.51		0.81	0.48–1.34	
	21+	218/84	1.06	0.73–1.54		0.84	0.52–1.36	
Fast food (g)	0	343/135	1		0.387	1		0.588
	1+	234/85	1.16	0.83–1.63		1.13	0.74–1.72	
Meat, poultry and organ meats (g)	<91	197/68	1		0.107	1		0.368
	91–150	199/68	1.05	0.70–1.58		1.30	0.78–2.17	
	150+	181/84	1.39	0.93–2.09		1.30	0.73–2.32	
Fish and seafood (g)	<43	189/76	1		0.911	1		0.991
	43–85	205/62	0.78	0.52–1.16		0.88	0.53–1.46	
	85+	183/82	1.02	0.70–1.50		1.00	0.61–1.64	
Fruits and dried fruits (g)	<132	185/80	1		**0.007**	1		**0.009**
	132–229	195/71	0.73	0.50–1.09		0.70	0.43–1.16	
	229+	197/69	0.57	0.38–0.86		0.50	0.30–0.84	
Grains and cereals (g)	<444	194/72	1		0.173	1		0.134
	444–637	196/69	0.80	0.53–1.19		0.85	0.50–1.42	
	637+	187/79	0.75	0.49–1.14		0.64	0.36–1.15	
Mushroom, fungi and nuts (g)	<2	183/84	1		**0.037**	1		0.168
	2–13	189/71	0.79	0.54–1.16		0.83	0.50–1.36	
	13+	205/65	0.66	0.44–0.98		0.70	0.42–1.16	
Soy and soy products (g)	<12	193/73	1		0.731	1		0.550
	12–54	188/77	1.05	0.71–1.55		1.15	0.70–1.89	
	54+	196/70	0.93	0.63–1.38		0.86	0.52–1.42	
Vegetables and legumes (g)	<119	185/80	1		**0.009**	1		**0.013**
	119–207	190/76	0.86	0.58–1.27		0.79	0.48–1.29	
	207+	202/64	0.58	0.38–0.87		0.51	0.30–0.87	
Milk and milk products (g)	<4	179/80	1		0.054	1		0.624
	4–30	193/83	1.08	0.74–1.58		1.53	0.94–2.49	
	30+	205/57	0.67	0.45–1.01		1.14	0.68–1.91	
Tea and coffee (ml)	<179	194/64	1		0.166	1		0.671
	179–464	209/65	0.83	0.55–1.24		0.58	0.34–0.97	
	464+	174/91	1.32	0.89–1.96		1.12	0.68–1.84	
% energy from carbohydrate	—-	—-	0.98	0.96–1.00	**0.013**	0.98	0.95–1.00	0.067
% energy from protein	—-	—-	1.03	0.98–1.08	0.211	1.00	0.95–1.06	0.921
% energy from total fat	—-	—-	1.02	1.00–1.04	0.130	1.03	1.00–1.06	0.089
% energy from saturated fat	—-	—-	1.03	0.95–1.13	0.436	1.02	0.92–1.14	0.714
Cholesterol (mg)	—-	—-	1.00	0.99–1.00	0.275	1.00	0.99–1.00	0.963
Dietary fiber (g)	—-	—-	0.97	0.94–1.00	**0.036**	0.96	0.93–1.00	0.054
Vitamin C (mg)	—-	—-	0.40	0.19–0.85	**0.017**	0.36	0.14–0.89	**0.028**

^1^ Tertiles based on the distribution of all subjects, except two categories for the fast food intake (intake = 0 vs. intake > 0 g per day)

^2^ Further adjusted for BMI, current smoker status (yes/no), current drinker status (yes/no), central obesity (yes/no), triglyceride >1.7 mmol/l (yes/no), reduced HDL-cholesterol (yes/no), hypertension (yes/no), impaired fasting glucose or diabetes (yes/no), and the *PNPLA3* genotypes (CC vs. CG vs. GG genotypes). Energy intake was further adjusted in the multivariate model of food group intake analysis.

^3^ *P*_*trend*_ was examined by entering tertiles of food group intake as a fixed factor for the models of food group intake analysis, whereas *P* value was used for the models of nutrient intake analysis.

^4^ Included mainly sweetened beverages or juice, carbonated drinks and sports drinks

NAFLD, non-alcoholic fatty liver disease

## Discussion

In this cross-sectional, population based study, lower DQI-I was associated with increased likelihood of having NAFLD in Hong Kong Chinese adults. The association was stronger in male participants compared to female participants, and in individuals who were overweight or obese compared to those were normal weight. Our study also suggested that a higher intake of vegetables and legumes, fruits and dried fruits, as well as vitamin C was associated with a reduced likelihood of having NAFLD.

To our knowledge, only few observational studies [24–29] have been conducted to examine the association between dietary patterns and NAFLD in adults and one prospective study has been done in a population-based cohort of adolescents [30]. While Hasehemi kani *et al*. reported that higher scores in four different diet-quality indices might protect against NAFLD in a group of Iranian adults attending a gastrointestinal research clinic [24], Kongtogianni *et al*. found that greater adherence to the Mediterranean diet as measured using the MDS was associated with less severity of fatty liver disease but was not associated with the likelihood of having NAFLD [25]. A recent cross-sectional, population based study, however, showed that a dietary pattern, characterized by increased intake of alcohol and meat (poultry), and reduced consumption of tea, was associated with higher liver fat content in adults [29]. Only one observational study has previously investigated exploratory dietary patterns and NAFLD risk, which was limited to adolescents [30]. A Western dietary pattern characterized by a high intake of takeaway foods, confectionary, red meat, refined grains, processed meats, chips, sauces, full-fat dairy products, and soft drinks at 14 years of age was prospectively associated with NAFLD at 17 years. Different study design, choices and assessment methods of outcome measures, covariates and confounding factors included in the statistical analysis, as well as methods to generate dietary quality indices or dietary pattern scores in various studies might lead to the mixed findings.

Previous studies have related DQI-I or MDS with cardiovascular risk, obesity and metabolic outcomes, and generally support that better diet quality is associated with better cardiovascular or metabolic outcomes [50,51]. To our knowledge, our study is the first study to report a positive association between DQI-I and NAFLD prevalence. Our findings also suggested that subjects consuming vegetables and legumes, as well as fruits and dried fruits of approximately ≥ 200 g/day each were likely to have 50% reduction in the likelihood of having NAFLD in comparison to subjects in the lowest tertile of consumption of these food groups. These intake levels are in line with the recommended intake of at least 400 g/day of vegetables and fruits by the World Health Organization for the prevention of diet-related chronic diseases [52]. Contrary to our findings that no association was observed between MDS and NAFLD prevalence, several observational studies and clinical trials have preliminarily suggested that higher adherence to the Mediterranean diet might protect against NAFLD [31,32]. Although the Chinese diet has many similar features with the Mediterranean diet, in that vegetable and fruit consumption is high, and fat and meat consumption is low, the consumption of legumes, milk and milk products, nuts, olive oil and wine was less in our cohort than the traditional Mediterranean diet. These differences may be one of the reasons to explain the absence of association between MDS and NAFLD prevalence. Moreover, the various scores differ in many aspects of both indices, such as the items included, the cut-off values used, and the exact method of scoring may be some reasons to explain the different results of DQI-I and MDS in the present study. For example, the scores of various components of the DQI-I are based on recommended reference intake that are beneficial for health, whereas the cut-off value of each component in the MDS is based on the group median intake of each component. The latter scoring system may lead to bias because the MDS calculation is based on cohort- and sex-specific median values across eight food categories of the studied sample, and it may not be related to a healthy level of intake *per se* [53].

Our data showed that the association between both diet-quality scores and the prevalence of NAFLD was in general stronger in male than in female, and in overweight or obese subjects than in normal weight subjects. These observations were different from those reported by Koch *et al*., in which no significant interaction with age, sex, BMI, or type 2 diabetes status was detected between dietary pattern scores and liver fat measured using magnetic resonance imaging as liver signal intensity [29]. The gender- and BMI-specific differences in the association between diet-quality scores and the prevalence of NAFLD in our study could be largely explained by the differences in metabolic factors. Our data also suggested that the association between both diet-quality scores and the prevalence of NAFLD was not affected by the *PNPLA3* genotypes. Previous studies focusing on the interaction between the PNPLA3 genotypes and single nutrient intake, such as dietary sugar and essential omega polyunsaturated fatty acids showed that the association of these nutrients with NAFLD might be driven by a predisposing GG genotype [54,55].

The strengths of our study include relatively large sample size, inclusion of subjects from the general population, and the use of ^1^H-MRS to assess hepatic steatosis. However, our study has several limitations. Our study was of cross-sectional in nature, thus it was not possible to examine the causal relationship between diet-quality scores and the likelihood of having NAFLD. Moreover, the FFQ captured only the short term dietary and food intakes of the subjects. Examining the relationship between diet and risk of chronic diseases using longer-term dietary and food intake data are more useful. Besides, although various common factors and major medical conditions have been adjusted in the analysis, residual potential confounding from other lifestyle factors related to the development of NAFLD, such as physical activity level might still exist [56],

## Conclusions

A better diet quality as characterized by a higher DQI-I and a higher consumption of vegetables, legumes and fruits was associated with a reduced likelihood of having NAFLD in Chinese adults in Hong Kong.

## Supporting Information

S1 File Details of the study. (PDF)

S2 File Study protocol of the cross-sectional population screening NAFLD study. (DOC)

## Abbreviations

BMI: body mass index
DBP: diastolic blood pressure
DM: diabetes mellitus
DQI-I: Diet Quality Index-International
FFQ: food frequency questionnaire
^1^H-MRS: proton-magnetic resonance spectroscopy
HC: hip circumference
IHTG: intrahepatic triglyceride content
MDS: Mediterranean Diet Score
NAFLD: nonalcoholic fatty liver disease
NASH: nonalcoholic steatohepatitis
SBP: systolic blood pressure
WC: waist circumference
