# Supplementary material for: Diet-Quality Scores and Prevalence of Nonalcoholic Fatty Liver Disease: A Population Study Using Proton-Magnetic Resonance Spectroscopy
Source: PLoS One. 2015 Sep 29;10(9):e0139310. doi: 10.1371/journal.pone.0139310 (PMC4587971; doi:10.1371/journal.pone.0139310)
Supplement: S1 File — (PDF) [file pone.0139310.s001.pdf]

Downloaded from http://gut.bmj.com/ on June 14, 2015 - Published by group.bmj.com

ORIGINAL ARTICLE

Prevalence of non-alcoholic fatty liver disease and advanced fibrosis in Hong Kong Chinese: a population study using proton-magnetic resonance spectroscopy and transient elastography

Vincent Wai-Sun Wong,[1,2] Winnie Chiu-Wing Chu,[1,3] Grace Lai-Hung Wong,[1,2] Ruth Suk-Mei Chan,[4] Angel Mei-Ling Chim,[1,2] Arlinking Ong,[1,2,5] David Ka-Wai Yeung,[6] Karen Kar-Lum Yiu,[1,2] Shirley Ho-Ting Chu,[1,2] Jean Woo,[2,4] Francis Ka-Leung Chan,[1,2] Henry Lik-Yuen Chan[1,2]

[1]Institute of Digestive Disease, The Chinese University of Hong Kong, Hong Kong, China
[2]Department of Medicine and Therapeutics, The Chinese University of Hong Kong, Hong Kong, China
[3]Department of Imaging and Interventional Radiology, The Chinese University of Hong Kong, Hong Kong, China
[4]Centre for Nutritional Studies, The Chinese University of Hong Kong, Hong Kong, China
[5]Department of Medicine, Section of Gastroenterology, University of Santo Tomas Hospital, Espana Manila, Philippines
[6]Department of Clinical Oncology, The Chinese University of Hong Kong, Hong Kong, China

Correspondence to
Dr Henry Lik-Yuen Chan, Department of Medicine and Therapeutics, Prince of Wales Hospital, 30—32 Ngan Shing Street, Shatin, Hong Kong, China; hlychan@cuhk.edu.hk

Dr Winnie Chiu-Wing Chu, Department of Imaging and Interventional Radiology, Prince of Wales Hospital, 30—32 Ngan Shing Street Shatin, Hong Kong, China; winnie@med.cuhk.edu.hk

Revised 30 May 2011
Accepted 10 June 2011
Published Online First 16 August 2011

ABSTRACT

Objective Knowledge of the epidemiology of non-alcoholic fatty liver disease (NAFLD) is incomplete because liver biopsy cannot be performed on the general population to assess disease severity. New non-invasive tests allow accurate and safe assessment in healthy individuals. The aim of this study was to examine the prevalence of NAFLD and advanced fibrosis in the general Hong Kong Chinese population.

Methods Subjects were recruited from the community by random selection from the government census database. Liver fat and fibrosis were assessed by proton-magnetic resonance spectroscopy and transient elastography, respectively.

Results Overall, 264 of 922 (28.6%) subjects had intrahepatic triglyceride content ≥5%. Excluding 12 subjects with significant alcohol consumption, the population prevalence of NAFLD was 27.3% (95% CI 24.5% to 30.2%). Each component of the metabolic syndrome increased the risk of fatty liver in a dose-dependent manner (prevalence of 4.5% in subjects without any component and 80.0% in those with all five components). 8 (3.7%) patients with fatty liver had liver stiffness ≥9.6 kPa, a level suggestive of advanced fibrosis. Body mass index and alanine aminotransferase level were independent factors associated with liver stiffness. Together with other clinical prediction scores, the estimated prevalence of advanced fibrosis in patients with fatty liver in the community was <10%. Compared with non-drinkers, modest drinkers (<10 g per day) did not have higher risk of fatty liver after adjustment for demographic and metabolic factors. The liver stiffness was 4.7±1.9 kPa in modest drinkers and 4.6±1.7 kPa in non-drinkers (p=0.54).

Conclusion NAFLD is found in over a quarter of the general adult Chinese population, but the proportion of patients with advanced fibrosis is low. Modest alcohol consumption does not increase the risk of fatty liver or liver fibrosis.

Significance of this study

What is already known about this subject?

- Non-alcoholic fatty liver disease (NAFLD) is one of the most common chronic liver diseases. Although Asia harbours the highest number of diabetic and obese patients, epidemiological data on NAFLD are scarce.
- Few studies have evaluated the severity of NAFLD in the general population. New non-invasive tests such as proton-magnetic resonance spectroscopy and transient elastography allow relatively accurate estimation of hepatic steatosis and fibrosis in the community.

What are the new findings?

- The population prevalence of NAFLD in Hong Kong Chinese was 27.3%.
- All components of the metabolic syndrome had an independent association with fatty liver.
- Around 4% of subjects with fatty liver in the community had advanced fibrosis as estimated by transient elastography.
- Modest alcohol consumption of <10 g per day did not increase the risk of fatty liver and liver fibrosis.

How might it impact on clinical practice in the foreseeable future?

- The study provides important epidemiological data on NAFLD in the general population. Clinicians may counsel patients based on the study findings.

INTRODUCTION

Non-alcoholic fatty liver disease (NAFLD) is the most common cause of abnormal liver function tests worldwide.[1] It may progress to cirrhosis and hepatocellular carcinoma,[2 3] and is believed to be the leading aetiology for cryptogenic cirrhosis.[4] NAFLD is strongly associated with obesity and the metabolic syndrome.[5 6] As a result, patients with NAFLD have increased mortality from cardiovascular diseases and malignancy in the long run.[7–10]

At present, knowledge of the epidemiology of NAFLD is incomplete. Histological cohorts represent highly selected patients and cannot reflect the situation in the general population.[11] Most population studies used abdominal ultrasonography for

Downloaded from http://gut.bmj.com/ on June 14, 2015 - Published by group.bmj.com

screening.[12–16] However, ultrasonography is operator dependent and is insensitive to mild hepatic steatosis.[17] In addition, the disease activity of NAFLD relies on histological assessment and is seldom assessed in population studies. In particular, Asia is expected to host the highest number of patients with diabetes and metabolic syndrome owing to its huge population and lifestyle changes.[18] Epidemiological data from this region are nevertheless scarce.[13 14]

In recent years, a number of non-invasive tests for liver diseases have been developed. Proton-magnetic resonance spectroscopy (^{1}H-MRS) quantifies hepatic steatosis by measuring proton signals from the acyl groups of hepatocyte triglyceride stores.[19] The measurement is reproducible and correlates well with the degree of hepatic steatosis by histology.[20–22] Transient elastography by Fibroscan (Echosens, Paris, France) estimates liver fibrosis by measuring liver stiffness. The measurement has good correlation with histological liver fibrosis in patients with different liver diseases.[23 24] Its performance has also been validated in patients with NAFLD, and is not affected by the degree of hepatic steatosis and body adiposity.[25 26] These new tests make population screening possible.

In this study, we aimed to study the prevalence of NAFLD and advanced liver fibrosis in the general Chinese population by ^{1}H-MRS and transient elastography, respectively. We also aimed to study factors associated with NAFLD.

METHODS

Patients

This was a cross-sectional study. Subjects from the general population were randomly selected from the census database of the Hong Kong Government, and were invited by mail and phone calls.[27] We included subjects aged 18–70 years. Subjects with active malignancy, metallic implants or other contraindications to MRI, positive hepatitis B surface antigen or antibody against hepatitis C virus, secondary causes of fatty liver (eg, consumption of amiodarone and tamoxifen) and decompensated liver disease (defined as bilirubin >50 μmol/l, albumin <35 g/l, platelet count $<150\times10^9$/l, international normalised ratio >1.3, or the presence of ascites or varices) were excluded. The study protocol was approved by the Clinical Research Ethics Committee of The Chinese University of Hong Kong. All subjects provided informed written consent.

Clinical assessment

At the clinic visit, drug history, alcohol intake, smoking and past medical history were recorded using a standardised questionnaire. Alcohol consumption of 20 g daily (140 g weekly) for men and 10 g daily (70 g weekly) for women was the threshold to define NAFLD.[28] Anthropometric measurements included body weight, body height and waist circumference. Body mass index (BMI) was calculated as weight (kg) divided by height (m^2). Waist circumference was measured at a level midway between the lower rib margin and iliac crest with the tape all around the body in the horizontal position. Blood tests including liver biochemistry, glucose and lipids were performed after fasting for 8 h.

Metabolic syndrome was defined according to the ethnic-specific criteria by the International Diabetes Federation, which was modified from the National Cholesterol Education Program, Adult Treatment Panel III Guidelines, as any three of the following: (1) central obesity (waist circumference ≥90 cm in men and ≥80 cm in women); (2) triglycerides >1.7 mmol/l; (3) reduced high-density lipoprotein-cholesterol (<1.03 mmol/l in men and <1.29 mmol/l in women); (4) blood pressure ≥130/85 mm Hg; and (5) fasting plasma glucose ≥5.6 mmol/l; or receiving treatment for the above metabolic abnormalities.[29]

The degree of liver fibrosis was estimated by transient elastography and clinical prediction formulae. The aspartate aminotransferase (AST) to alanine aminotransferase (ALT) level was calculated as AST/ALT. The AST to platelet ratio index (APRI) was calculated as AST (/upper limit of normal)/platelet count $(\times10^9/\text{l})\times100$.[30] FIB-4 was calculated as age × AST (IU/l)/platelet count $(\times10^9/\text{l})\times\sqrt{\text{ALT}}$ (U/l).[31] The NAFLD fibrosis score was calculated as −1.675+0.037 × age (years) +0.094 × BMI (kg/m^2) +1.13× impaired fasting glucose/diabetes (yes=1, no=0) +0.99× AST/ALT ratio−0.013× platelet count $(\times10^9/\text{l})$−0.66× albumin (g/dl).[32 33] The BARD score was the weighted sum of three variables (BMI ≥28 kg/m^2=1 point, AST/ALT ratio ≥0.8=2 points, diabetes=1 point).[34]

Proton-magnetic resonance spectroscopy

^{1}H-MRS was performed to measure intrahepatic triglyceride (IHTG) content within 8 weeks from the baseline visit. A whole-body 3.0 T scanner with a single voxel point-resolved spectroscopy sequence and an echo time of 40 ms and repetition time of 5000 ms was used. A survey scan was first performed in the abdominal region to help in positioning a volume measuring 20 (AP) ×15 (RL) ×40 (FH) mm within the liver. The scanner's built-in body coil was used for both signal transmission and reception. A no-water-suppressed spectrum was acquired using 32 signal averages and the data were exported for offline spectral analysis. Water (4.65 ppm) and lipid (1.3 ppm) peak amplitudes were measured to determine vertebral marrow fat content, which was defined as the relative fat signal amplitude in terms of a percentage of the total signal amplitude (water and fat) and calculated according to the following equation: fat content=$(I_{\text{fat}}/(I_{\text{fat}}+I_{\text{water}}))\times100$, where I_{fat} and I_{water} are the peak amplitudes of fat and water, respectively. Correction for relaxation loss was not applied because of the relatively long repetition time and short echo time. An IHTG content of 5% was used to distinguish between patients with and without fatty liver.[35]

Transient elastography

Liver stiffness measurement by transient elastography was performed during the baseline clinic visit according to the instructions and training provided by the manufacturer. Measurements were performed on the right lobe of the liver through intercostal spaces with the subject lying in dorsal decubitus with the right arm in maximal abduction. Ten successful acquisitions were performed on each subject. The success rate was calculated as the ratio of the number of successful acquisitions over the total number of acquisitions. The median value represented the liver elastic modulus. Liver stiffness measurements were considered reliable only if 10 successful acquisitions were obtained, the success rate was >60% and the interquartile range to median ratio of the 10 acquisitions was <0.3. Liver stiffness was expressed in kiloPascal (kPa). Operators must have performed at least 50 procedures before participating in this study, and were blinded to all clinical data and the diagnoses of the patients. The cut-off value of 9.6 kPa was used to estimate the number of subjects with advanced fibrosis. This cut-off value had specificity >90% in a previous validation study using liver histology as the reference standard.[26]

Statistical analysis

Statistical tests were performed using the Statistical Package for Social Sciences version 16.0. Continuous variables were expressed in mean±SD and compared using the unpaired t test if

Gut 2012;61:409–415. doi:10.1136/gutjnl-2011-300342

Downloaded from http://gut.bmj.com/ on June 14, 2015 - Published by group.bmj.com

they were normally distributed. Skewed variables were expressed as median (range) and compared using the Mann–Whitney U test. Q–Q plots were used to screen for normal distribution. Categorical variables were compared using χ^2 test or Fisher exact test as appropriate. Binary logistic regression analysis was performed to identify factors associated with fatty liver. The Spearman correlation test was performed to assess the correlation between clinical factors and liver stiffness in patients with fatty liver. Linear regression analysis was performed to identify independent factors associated with liver stiffness.

According to the census in 2006, the Hong Kong population aged ≥15 years was 5 924 671. A sample size of 900 would determine the prevalence of NAFLD in Hong Kong at a 3% CI and 95% confidence level for a prevalence of 20–50%. Assuming 10% of the subjects would be excluded due to viral hepatitis and other reasons, a total sample size of 1000 subjects was required.

RESULTS

From May 2008 to September 2010, invitation letters were sent to 3000 randomly selected Hong Kong residents from the census database. One thousand and sixty-nine subjects responded, with a response rate of 35.6%. After excluding subjects with contraindications to or failed ^{1}H-MRS examination and those with concurrent viral hepatitis, 922 subjects were included in the final analysis (figure 1). The mean age of the subjects was 48±11 years (range 19–72 years), and 533 (57.8%) were women (table 1). A total of 48 (5.2%) and 143 (15.5%) subjects had diabetes and hypertension, respectively, and 209 (22.8%) subjects had BMI ≥25.0 kg/m^2. All subjects were ethnic Chinese.

Prevalence of fatty liver

Overall, 264 (28.6%) subjects had fatty liver detected by ^{1}H-MRS. Only 12 subjects with fatty liver had significant alcohol consumption between 140 and 350 g per week. After excluding these subjects, the population prevalence of NAFLD was 27.3% (95% CI 24.5% to 30.2%). Among subjects with fatty liver, 154 (58.3%) had an IHTG content of 5–10.9%, and 110 (41.7%) had an IHTG content ≥11.0%, a level suggestive of >33% hepatic steatosis by histology.[21]

The prevalence of fatty liver was 36.8% (95% CI 32.0% to 41.6%) in men and 22.7% (95% CI 19.1% to 26.3%) in women. In men, the prevalence of fatty liver peaked at 40 years of age and remained relatively constant up to the seventh decade (figure 2). In contrast, the prevalence of fatty liver remained low at 12–16% in women younger than 50 years, and increased steadily after the menopause.

By univariate analysis, older age, male gender, high plasma creatinine, ferritin and haemoglobin levels, and components of the metabolic syndrome were associated with fatty liver (table 2). By multivariate analysis, a high ferritin level and components of the metabolic syndrome remained as independent factors associated with fatty liver, while the association with age and male gender became insignificant and could be largely explained by the differences in metabolic factors.

All five components of the metabolic syndrome according to the International Diabetes Federation criteria were independently associated with fatty liver (table 2). Moreover, a dose–response relationship was observed. The prevalence of fatty liver was only 4.5% in subjects without any component of the metabolic syndrome but 80.0% in those with all five components (figure 3).

Prevalence of advanced fibrosis

All subjects underwent liver stiffness measurement by transient elastography. Sixty subjects did not have 10 successful acquisitions and 103 subjects had an interquartile range to median ratio of the 10 acquisitions ≥0.3. The liver stiffness measurements of 759 subjects with valid examination were analysed (figure 1). Subjects with a valid examination had lower BMI (22.6±3.4 kg/m^2 vs 23.7±3.9 kg/m^2; p=0.002) and waist circumference (81±10 cm vs 84±11 cm; p<0.001). On the other hand, the percentage of subjects with or without a fatty liver with valid examination was similar (82.2% vs 82.4%; p=0.95).

The liver stiffness was significantly higher in subjects with fatty liver than in controls (5.1±1.9 kPa vs 4.4±1.6 kPa; p<0.001) (table 1). Eight (3.7%) patients with fatty liver and 7 (1.3%) controls had liver stiffness ≥9.6 kPa, a level suggestive of advanced fibrosis (p=0.032).

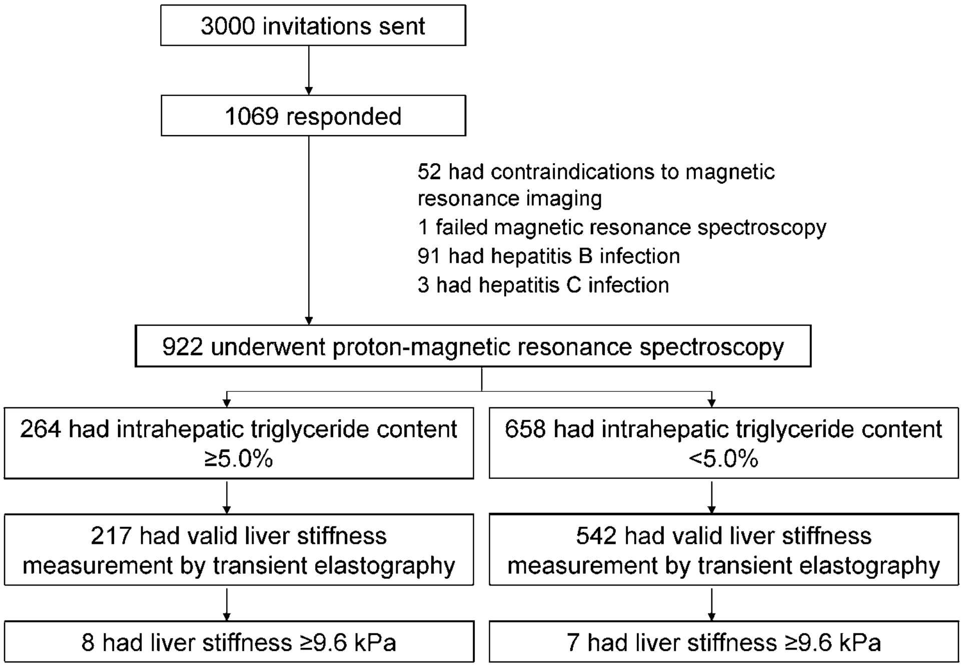

Figure 1 Study recruitment and participant flow.

Downloaded from http://gut.bmj.com/ on June 14, 2015 - Published by group.bmj.com

Table 1 Clinical characteristics of subjects with and without fatty liver

Characteristics	All	Fatty liver	No fatty liver	p Value
N	922	264	658	
Age (years)	48±11	51±9	47±11	<0.001
Male gender, n (%)	389 (42.2)	143 (54.2)	246 (37.4)	<0.001
Current smoker, n (%)	89 (9.7)	30 (11.4)	59 (9.0)	0.27
Current drinker, n (%)	203 (22.0)	76 (28.8)	127 (19.3)	0.002
Alcohol consumption (g/week)	0 (0–350)	0 (0–350)	0 (0–300)	0.006
Body mass index (kg/m^2)	22.8±3.5	25.3±3.4	21.8±3.0	<0.001
Men	23.3±3.0	25.0±2.8	22.4±2.6	<0.001
Women	22.4±3.8	25.8±4.0	21.4±3.1	<0.001
Waist circumference (cm)	81±10	89±8	79±9	<0.001
Men	85±9	90±7	83±8	<0.001
Women	79±10	88±8	76±9	<0.001
Waist-to-hip ratio	0.86±0.08	0.90±0.06	0.84±0.08	<0.001
Men	0.89±0.06	0.92±0.05	0.87±0.06	<0.001
Women	0.84±0.08	0.89±0.06	0.82±0.08	<0.001
Systolic blood pressure (mm Hg)	129±20	138±19	126±19	<0.001
Diastolic blood pressure (mm Hg)	82±12	88±12	79±11	<0.001
Creatinine (μmol/l)	75±15	78±16	74±15	<0.001
Total bilirubin (mmol/l)	14±6	13±5	14±6	0.32
Alkaline phosphatase (IU/l)	64±18	69±17	62±18	<0.001
Alanine aminotransferase (IU/l)	26±16	35±19	22±13	<0.001
Aspartate aminotransferase (IU/l)	21±12	24±10	20±13	<0.001
Fasting glucose (mmol/l)	5.1±0.9	5.6±1.4	5.0±0.6	<0.001
Haemoglobin A_{1c} (%)	5.4±0.6	5.8±0.9	5.3±0.4	<0.001
Total cholesterol (mmol/l)	5.2±1.0	5.4±1.0	5.1±1.0	<0.001
HDL-cholesterol (mmol/l)	1.5±0.4	1.3±0.3	1.6±0.4	<0.001
LDL-cholesterol (mmol/l)	3.0±0.9	3.2±0.9	2.9±0.8	<0.001
Triglycerides (mmol/l)	1.1 (0.3–21.3)	1.6 (0.3–21.3)	0.9 (0.3–9.1)	<0.001
Ferritin	352 (0–3449)	636 (5–3449)	286 (0–2909)	<0.001
Haemoglobin (g/dl)	13.8±1.5	14.3±1.4	13.6±1.5	<0.001
Diabetes, n (%)	48 (5.2)	29 (11.0)	19 (2.9)	<0.001
Hypertension, n (%)	143 (15.5)	81 (30.7)	62 (9.4)	<0.001
Metabolic syndrome, n (%)†	187 (20.3)	125 (47.3)	62 (9.4)	<0.001
Intrahepatic triglyceride content (%)	2.1 (0–44.2)	9.8 (5.0–44.2)	1.3 (0–4.9)	<0.001
Liver stiffness measurement (kPa)*	4.6±1.7	5.1±1.9	4.4±1.6	<0.001
Liver stiffness ≥9.6 kPa, n (%)*	15 (2.0)	8 (3.7)	7 (1.3)	0.032

*Included 759 subjects (217 with fatty liver and 542 without) with valid transient elastography examination.
†International Diabetes Federation criteria for metabolic syndrome.
HDL, high-density lipoprotein; LDL, low-density lipoprotein.
Continuous variables were expressed as mean±SD or median (range) as appropriate.

Among patients with fatty liver, liver stiffness had a positive correlation with obesity, systolic blood pressure, serum ALT and AST levels, and IHTG content. By multivariate analysis using a linear regression model, BMI (β 0.17; p=0.008) and ALT level (β 0.24; p=0.019) remained as independent factors associated with liver stiffness.

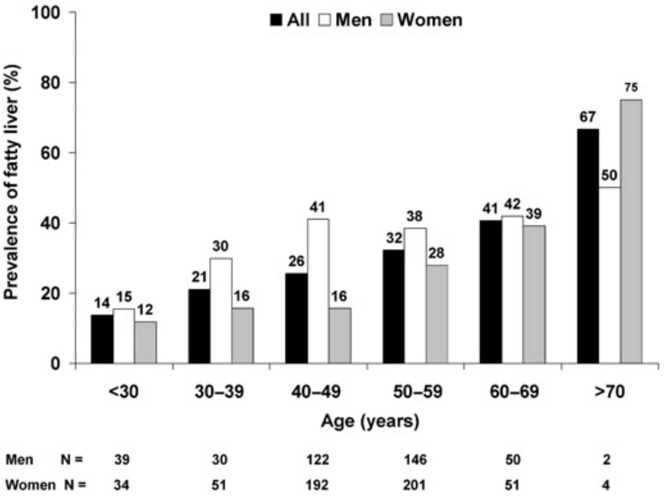

Figure 2 Prevalence of fatty liver by age and gender.

Based on the calculation of the 95th percentiles, 90% of subjects without fatty liver or other liver diseases had liver stiffness between 2.8 and 7.4 kPa, which reflected the normal range in the general population. The same normal range was found for both men and women when the analysis was stratified by gender.

Other than liver stiffness measurement, estimation of liver fibrosis was also performed using the AST/ALT ratio, APRI, FIB-4 score, NAFLD fibrosis score and BARD score. Based on different scores, the estimated prevalence of advanced fibrosis was between 0 and 12.1% in subjects with fatty liver (table 3). When lower cut-off values of the scores were used for screening, 83.7–95.5% of subjects with fatty liver were unlikely to have significant fibrosis.

Validation of the new ALT cut-off values

Four hundred and twenty-six subjects had elevated ALT according to the new cut-off values (≥30 IU/l in men and ≥19 IU/l in women), and 496 subjects had normal ALT. Fatty liver was found in 193 (45.3%) subjects with elevated ALT and

Gut 2012;61:409–415. doi:10.1136/gutjnl-2011-300342

Downloaded from http://gut.bmj.com/ on June 14, 2015 - Published by group.bmj.com

Table 2 Factors associated with fatty liver

Factors	Univariate analysis OR (95% CI)	p Value	Multivariate analysis OR (95% CI)	p Value
Age (years)	1.04 (1.02 to 1.05)	<0.001	1.00 (0.98 to 1.02)	0.78
Male gender	1.98 (1.48 to 2.64)	<0.001	1.03 (0.54 to 1.99)	0.93
Alcohol consumption (g/week)	1.003 (1.000 to 1.007)	0.077	0.99 (0.94 to 1.03)	0.55
Creatinine (μmol/l)	1.02 (1.01 to 1.03)	<0.001	1.00 (0.99 to 1.02)	0.64
Ferritin	1.001 (1.001 to 1.002)	<0.001	1.001 (1.001 to 1.001)	<0.001
Haemoglobin (g/dl)	1.40 (1.26 to 1.56)	<0.001	1.15 (0.98 to 1.34)	0.083
Central obesity*	4.63 (3.42 to 6.28)	<0.001	4.26 (2.91 to 6.24)	<0.001
Triglycerides >1.7 mmol/l*	6.19 (4.40 to 8.70)	<0.001	2.86 (1.88 to 4.35)	<0.001
Reduced HDL-cholesterol*	4.43 (3.02 to 6.51)	<0.001	2.54 (1.58 to 4.08)	<0.001
Impaired fasting glucose or diabetes*	3.69 (2.56 to 5.33)	<0.001	2.17 (1.39 to 3.40)	0.001
Hypertension*	3.36 (2.46 to 4.59)	<0.001	1.52 (1.02 to 2.26)	0.040

*International Diabetes Federation criteria for metabolic syndrome.
HDL, high-density lipoprotein.

71 (14.3%) subjects with normal ALT (p<0.001). The median IHTG content by ^{1}H-MRS was 4.3% (0–44.2%) in subjects with elevated ALT and 1.4% (0–35.1%) in subjects with normal ALT (p<0.001).

A dose–response relationship was observed between ALT level and the prevalence of fatty liver. Seventy-one of 496 (14.3%) subjects with ALT below the new cut-off values, 165 of 387 (42.6%) subjects with ALT between the new cut-off values and the old local laboratory cut-off value of 58 IU/l, and 28 of 39 (71.8%) subjects with ALT >58 IU/l had fatty liver (p<0.001). The median IHTG content of the three groups of subjects was 1.4% (0–35.1%), 4.1% (0–31.2%) and 11.6% (0.7–44.2%) (p<0.001 by Kruskal–Wallis test).

Among 759 subjects with valid liver stiffness measurements by transient elastography, 347 had elevated ALT and 412 had normal ALT. Nine (2.6%) subjects with elevated ALT and 6 (1.5%) subjects with normal ALT had liver stiffness ≥9.6 kPa (p=0.26). The liver stiffness was 4.8±1.8 kPa in subjects with elevated ALT and 4.5±1.7 kPa in subjects with normal ALT (p=0.013).

Effect of modest alcohol consumption

Seven hundred and twenty subjects (78.1%) were non-drinkers, 148 (16.1%) had modest alcohol consumption of <70 g per week, and 54 (5.9%) had alcohol consumption ≥70 g per week. The amount of alcohol consumption had a weak positive correlation with IHTG content by ^{1}H-MRS (Spearman correlation coefficient 0.11; p=0.001) but not liver stiffness (Spearman correlation coefficient 0.003; p=0.94). Fatty liver was detected in 56 (37.8%) modest drinkers and 190 (26.4%) non-drinkers (p=0.005). The median IHTG content was 2.7% (range 0.1–44.2%) in modest drinkers and 1.9% (range 0–35.1%) in non-drinkers (p=0.012). After adjustment for age, gender and metabolic syndrome, modest alcohol consumption was no longer associated with fatty liver (adjusted OR 1.37; 95% CI 0.89 to 2.11; p=0.15).

The proportion of subjects with liver stiffness ≥9.6 kPa was similar between modest drinkers (3 of 130 (2.3%)) and non-drinkers (10 of 583 (1.7%)) (p=0.72). The liver stiffness was 4.7±1.9 kPa in modest drinkers and 4.6±1.7 kPa in non-drinkers (p=0.54).

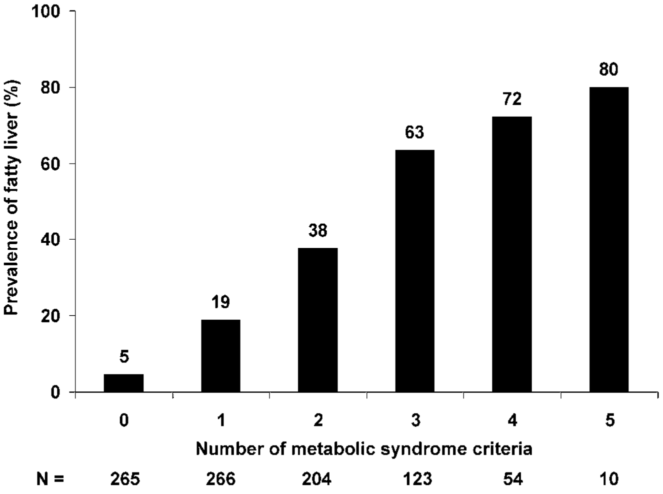

Figure 3 Prevalence of fatty liver in subjects harbouring different numbers of components of the metabolic syndrome according to the International Diabetes Federation criteria.

DISCUSSION

In this large epidemiological study in the general adult Chinese population, the prevalence of NAFLD is 27.3%. Around 4% of patients with fatty liver have advanced fibrosis. Fatty liver was found in 14.3% of subjects with normal ALT according to the updated stringent cut-off values (<30 IU/l in men and <19 IU/l in women). Modest alcohol consumption increases hepatic fat but does not increase the risk of advanced fibrosis.

Compared with previous reports, our study recruited asymptomatic individuals from the general population by using the government census database. The use of state-of-the-art

Table 3 Estimated prevalence of advanced fibrosis in 264 fatty liver subjects based on clinical prediction formulae

Formulae	Cut-off	n	95% CI
AST/ALT ratio	1.0	32 (12.1%)	8.2% to 16.1%
APRI*	0.5	12 (4.5%)	2.0% to 7.1%
	1.5	0	0 to 0
FIB-4*	1.30	26 (9.8%)	6.3% to 13.4%
	2.67	1 (0.4%)	0 to 1.1%
NAFLD fibrosis score*	−1.455	43 (16.3%)	0 to 20.7%
	0.676	0	0 to 0
BARD score	2	16 (6.1%)	3.2% to 8.9%

*The lower cut-off values had high sensitivity in excluding advanced fibrosis, while the higher cut-off values had high specificity in ruling in advanced fibrosis.
ALT, alanine aminotransferase; APRI, AST to platelet ratio index; AST, aspartate aminotransferase; NAFLD, non-alcoholic fatty liver disease.

Downloaded from http://gut.bmj.com/ on June 14, 2015 - Published by group.bmj.com

non-invasive tests allowed accurate assessment of hepatic steatosis and fibrosis in a large number of subjects in the community, in which liver biopsy may not be feasible.

Previous epidemiological studies reported a prevalence of NAFLD of 20–40% in the USA and Europe.[12 15 35 36] Owing to westernisation of lifestyle, obesity and metabolic syndrome are increasingly prevalent in Asia. A summary of Asia-Pacific studies showed that the prevalence of NAFLD was 16–42%.[37] However, many studies from Asia were limited by the lack of a uniform definition of NAFLD and the inclusion of hospital patients instead of community subjects. As such, our current study confirms that the prevalence of NAFLD in the Chinese population is similar to that in western countries.

The prevalence of NAFLD is higher in men and postmenopausal women. The underlying cause is not completely understood, but dysregulated sex hormones appear to affect the liver in various ways. For example, NAFLD is prevalent in women with polycystic ovary syndrome.[38] In patients with viral hepatitis, male gender is associated with disease progression and higher risk of hepatocellular carcinoma.[39 40] In line with previous histological series, metabolic syndrome has the strongest association with NAFLD.[5 6] Our current study suggests that all components of metabolic syndrome have independent and important association with NAFLD. It would be important to investigate the relative contribution by different metabolic factors to NAFLD progression in longitudinal studies.[2] In addition, ferritin is another independent factor associated with fatty liver. While ferritin is an acute phase protein that may be increased in a systemic inflammatory state, the interaction between iron stored and NAFLD has been underscored by various studies.[41 42]

Since ultrasound scan can only detect fatty liver but cannot assess disease severity, current knowledge of the impact of NAFLD is incomplete. Recently, Williams and colleagues reported a study of 328 patients at the Brooke Army Medical Center.[15] All patients with fatty liver detected by ultrasound scan were invited to have liver biopsy examination. Overall, 151 (46%) patients had fatty liver. Among 134 patients with fatty liver who underwent liver biopsy, 40 (30%) had non-alcoholic steatohepatitis and 9 (7%) had advanced fibrosis. Since patients in this study were recruited from the clinic, it was not actually a population study. The high prevalence of diabetes and obesity in this cohort also explains the high proportion of patients with NAFLD and steatohepatitis. Nevertheless, this is already one of the best studies addressing the prevalence and severity of NAFLD in a primary care setting. With the use of ^{1}H-MRS and transient elastography, our current study was able to assess both hepatic steatosis and fibrosis. Based on liver stiffness measurements and confirmed by various clinical prediction models of liver fibrosis, the prevalence of advanced fibrosis in patients with fatty liver is ∼4% and unlikely to exceed 10%. Thus, at most, 3% of the general population would have advanced fibrosis secondary to fatty liver.

Since the population prevalence of NAFLD is high, further studies should be conducted to determine the value of ^{1}H-MRS screening for at-risk groups such as those with metabolic syndrome. Unlike full MRI, ^{1}H-MRS can be done within 20 min. With development of new technologies, the test may even be combined with magnetic resonance elastography for liver fibrosis assessment and phosphorus-MRS for liver injury. This may provide clinicians and patients with a comprehensive but non-invasive evaluation of the liver.

Recently, it was recognised that old ALT cut-offs tend to be too high because the normal range was derived from healthy volunteers who might harbour fatty liver. A set of updated ALT cut-off values (30 IU/l in men and 19 IU/l in women) was proposed to improve the accuracy in detecting patients with liver diseases.[43] In fact, patients with high-normal ALT are at increased risk of liver-related mortality in the long run.[44] In our study, we confirmed that subjects with normal ALT according to the updated cut-offs were less likely to have fatty liver. However, ALT only had a weak correlation with liver stiffness. In histological series of patients with NAFLD, a normal ALT level could not reliably exclude steatohepatitis and advanced fibrosis.[45 46] Patients progressing to cirrhosis may even have burnt-out disease and a paradoxical drop in ALT level.[47] Therefore, while the prevalence of NAFLD is generally lower in people with normal ALT, ALT is a poor marker of disease severity in patients with NAFLD.

Modest alcohol consumption protects the cardiovascular system. Its effect on fatty liver is unclear. A posthoc analysis of the Third National Health and Nutrition Examination Survey suggested that modest wine drinkers (<10 g per day) had lower risk of fatty liver.[48] However, that study used elevated ALT to define fatty liver and no imaging studies were performed. In our study, even modest drinkers had a higher prevalence of fatty liver, although the effect appears to be less important after adjustment for other metabolic factors. Moreover, our data clearly demonstrate that modest drinking does not increase the risk of advanced fibrosis.

Our study has a few limitations. First, all subjects were ethnic Chinese with a low prevalence of excessive alcohol consumption. Our findings cannot reflect the situation in different countries. However, the case recruitment from a government census database and the use of ^{1}H-MRS and transient elastography made this one of the few studies in the literature that provide comprehensive epidemiological data on NAFLD. Secondly, while transient elastography has excellent negative predictive value in excluding advanced fibrosis in patients with NAFLD, the positive predictive value in diagnosing the condition is imperfect.[26] Therefore, we minimised the bias by supplementing the data with five other clinical prediction scores. The results consistently confirmed a low rate of advanced fibrosis in patients with NAFLD in the general population. Thirdly, IHTG was measured once and we cannot exclude the possibility that the value may change with time. However, a previous study in college students showed that while a short-term increase in food intake might change the metabolic profile and ALT levels, the effect on IHTG was modest.[49] The small variability in IHTG would be unlikely to affect the major observations in this study. Finally, we did not exclude rarer liver diseases such as autoimmune hepatitis, primary biliary cirrhosis and metabolic liver diseases. However, the population prevalence of these diseases is low and would be unlikely to affect the results significantly. Similarly, the prevalence of non-alcoholic steatohepatitis is unknown as liver biopsy was not performed. However, recent data suggest that liver fibrosis is more important than other histological features of steatohepatitis in determining the long-term liver-related mortality of patients with NAFLD.[50]

In conclusion, NAFLD is found in over a quarter of the general adult Chinese population, but the proportion of patients with advanced fibrosis is low. Modest alcohol consumption increases the risk of fatty liver but does not affect liver fibrosis.

Acknowledgements We would like to thank the following students and research assistants for helping with data collection: Andrew Hayward, Catherine Hayward, Mandy Law, Mia Li and April Wong.

Downloaded from http://gut.bmj.com/ on June 14, 2015 - Published by group.bmj.com

Funding The study was supported by a grant from the Health and Health Services Research Fund sponsored by the Government of Hong Kong SAR (Reference number 07080081).

Competing interests VWW and GLW have received paid lecture fees from Echosens. HL-KC is an advisory board member of Echosens.

Ethics approval This study was approved by the Clinical Research Ethics Committee, The Chinese University of Hong Kong.

Contributors Study conception, VWW, WCC, HLC; data collection, VWW, WCC, GLW, RSC, AMC, AO, DKY, KKY, SHC; data analysis, VWW, HLC; administrative support, JW, FKC, HLC; manuscript drafting, VWW, WCC, HLC. All authors read and approved the final version of the manuscript.

Provenance and peer review Not commissioned; externally peer reviewed.

REFERENCES

1. **Chitturi S,** Wong VW, Farrell G. Nonalcoholic fatty liver in Asia: firmly entrenched and rapidly gaining ground. *J Gastroenterol Hepatol* 2011;**26**(Suppl 1):163—72.
2. **Wong VW,** Wong GL, Choi PC, *et al*. Disease progression of non-alcoholic fatty liver disease: a prospective study with paired liver biopsies at 3 years. *Gut* 2010;**59**:969—74.
3. **Ascha MS,** Hanouneh IA, Lopez R, *et al*. The incidence and risk factors of hepatocellular carcinoma in patients with nonalcoholic steatohepatitis. *Hepatology* 2010;**51**:1972—8.
4. **Caldwell SH,** Oelsner DH, Iezzoni JC, *et al*. Cryptogenic cirrhosis: clinical characterization and risk factors for underlying disease. *Hepatology* 1999;**29**:664—9.
5. **Wong VW,** Hui AY, Tsang SW, *et al*. Metabolic and adipokine profile of Chinese patients with nonalcoholic fatty liver disease. *Clin Gastroenterol Hepatol* 2006;**4**:1154—61.
6. **Wong VW,** Hui AY, Tsang SW, *et al*. Prevalence of undiagnosed diabetes and postchallenge hyperglycaemia in Chinese patients with non-alcoholic fatty liver disease. *Aliment Pharmacol Ther* 2006;**24**:1215—22.
7. **Adams LA,** Lymp JF, St Sauver J, *et al*. The natural history of nonalcoholic fatty liver disease: a population-based cohort study. *Gastroenterology* 2005;**129**:113—21.
8. **Ekstedt M,** Franzen LE, Mathiesen UL, *et al*. Long-term follow-up of patients with NAFLD and elevated liver enzymes. *Hepatology* 2006;**44**:865—73.
9. **Rafiq N,** Bai C, Fang Y, *et al*. Long-term follow-up of patients with nonalcoholic fatty liver. *Clin Gastroenterol Hepatol* 2009;**7**:234—8.
10. **Wong VW,** Wong GL, Yip GW, *et al*. Coronary artery disease and cardiovascular outcomes in patients with non-alcoholic fatty liver disease. *Gut* 2011;**60**:1721—7.
11. **Wong VW,** Chan HL, Hui AY, *et al*. Clinical and histological features of non-alcoholic fatty liver disease in Hong Kong Chinese. *Aliment Pharmacol Ther* 2004;**20**:45—9.
12. **Bellentani S,** Saccoccio G, Masutti F, *et al*. Prevalence of and risk factors for hepatic steatosis in Northern Italy. *Ann Intern Med* 2000;**132**:112—17.
13. **Fan JG,** Zhu J, Li XJ, *et al*. Prevalence of and risk factors for fatty liver in a general population of Shanghai, China. *J Hepatol* 2005;**43**:508—14.
14. **Hamaguchi M,** Kojima T, Takeda N, *et al*. The metabolic syndrome as a predictor of nonalcoholic fatty liver disease. *Ann Intern Med* 2005;**143**:722—8.
15. **Williams CD,** Stengel J, Asike MI, *et al*. Prevalence of nonalcoholic fatty liver disease and nonalcoholic steatohepatitis among a largely middle-aged population utilizing ultrasound and liver biopsy: a prospective study. *Gastroenterology* 2011;**140**:124—31.
16. **Ayonrinde OT,** Olynyk JK, Beilin LJ, *et al*. Gender-specific differences in adipose distribution and adipocytokines influence adolescent nonalcoholic fatty liver disease. *Hepatology* 2011;**53**:800—9.
17. **Saadeh S,** Younossi ZM, Remer EM, *et al*. The utility of radiological imaging in nonalcoholic fatty liver disease. *Gastroenterology* 2002;**123**:745—50.
18. **Chan JC,** Malik V, Jia W, *et al*. Diabetes in Asia: epidemiology, risk factors, and pathophysiology. *JAMA* 2009;**301**:2129—40.
19. **Johnson NA,** Walton DW, Sachinwalla T, *et al*. Noninvasive assessment of hepatic lipid composition: advancing understanding and management of fatty liver disorders. *Hepatology* 2008;**47**:1513—23.
20. **Szczepaniak LS,** Nurenberg P, Leonard D, *et al*. Magnetic resonance spectroscopy to measure hepatic triglyceride content: prevalence of hepatic steatosis in the general population. *Am J Physiol Endocrinol Metab* 2005;**288**:E462—8.
21. **McPherson S,** Jonsson JR, Cowin GJ, *et al*. Magnetic resonance imaging and spectroscopy accurately estimate the severity of steatosis provided the stage of fibrosis is considered. *J Hepatol* 2009;**51**:389—97.
22. **Lee SS,** Park SH, Kim HJ, *et al*. Non-invasive assessment of hepatic steatosis: prospective comparison of the accuracy of imaging examinations. *J Hepatol* 2010;**52**:579—85.
23. **Wong GL,** Wong VW, Choi PC, *et al*. Assessment of fibrosis by transient elastography compared with liver biopsy and morphometry in chronic liver diseases. *Clin Gastroenterol Hepatol* 2008;**6**:1027—35.
24. **Wong VW,** Chan HL. Transient elastography. *J Gastroenterol Hepatol* 2010;**25**:1726—31.
25. **Nobili V,** Vizzutti F, Arena U, *et al*. Accuracy and reproducibility of transient elastography for the diagnosis of fibrosis in pediatric nonalcoholic steatohepatitis. *Hepatology* 2008;**48**:442—8.
26. **Wong VW,** Vergniol J, Wong GL, *et al*. Diagnosis of fibrosis and cirrhosis using liver stiffness measurement in nonalcoholic fatty liver disease. *Hepatology* 2010;**51**:454—62.
27. **Wong VW,** Wong GL, Tsang SW, *et al*. High prevalence of colorectal neoplasm in patients with non-alcoholic steatohepatitis. *Gut* 2011;**60**:829—36.
28. **Farrell GC,** Chitturi S, Lau GK, *et al*. Guidelines for the assessment and management of non-alcoholic fatty liver disease in the Asia-Pacific region: executive summary. *J Gastroenterol Hepatol* 2007;**22**:775—7.
29. **Alberti KG,** Eckel RH, Grundy SM, *et al*. Harmonizing the metabolic syndrome: a joint interim statement of the International Diabetes Federation Task Force on Epidemiology and Prevention; National Heart, Lung, and Blood Institute; American Heart Association; World Heart Federation; International Atherosclerosis Society; and International Association for the Study of Obesity. *Circulation* 2009;**120**:1640—5.
30. **Wai CT,** Greenson JK, Fontana RJ, *et al*. A simple noninvasive index can predict both significant fibrosis and cirrhosis in patients with chronic hepatitis C. *Hepatology* 2003;**38**:518—26.
31. **Sterling RK,** Lissen E, Clumeck N, *et al*; APRICOT Clinical Investigators. Development of a simple noninvasive index to predict significant fibrosis in patients with HIV/HCV coinfection. *Hepatology* 2006;**43**:1317—25.
32. **Angulo P,** Hui JM, Marchesini G, *et al*. The NAFLD fibrosis score: a noninvasive system that identifies liver fibrosis in patients with NAFLD. *Hepatology* 2007;**45**:846—54.
33. **Wong VW,** Wong GL, Chim AM, *et al*. Validation of the NAFLD fibrosis score in a Chinese population with low prevalence of advanced fibrosis. *Am J Gastroenterol* 2008;**103**:1682—8.
34. **Harrison SA,** Oliver D, Arnold HL, *et al*. Development and validation of a simple NAFLD clinical scoring system for identifying patients without advanced disease. *Gut* 2008;**57**:1441—7.
35. **Browning JD,** Szczepaniak LS, Dobbins R, *et al*. Prevalence of hepatic steatosis in an urban population in the United States: impact of ethnicity. *Hepatology* 2004;**40**:1387—95.
36. **Lonardo A,** Bellini M, Tartoni P, *et al*. The bright liver syndrome. Prevalence and determinants of a 'bright' liver echopattern. *Ital J Gastroenterol Hepatol* 1997;**29**:351—6.
37. **Amarapurkar DN,** Hashimoto E, Lesmana LA, *et al*. How common is non-alcoholic fatty liver disease in the Asia-Pacific region and are there local differences? *J Gastroenterol Hepatol* 2007;**22**:788—93.
38. **Cerda C,** Perez-Ayuso RM, Riquelme A, *et al*. Nonalcoholic fatty liver disease in women with polycystic ovary syndrome. *J Hepatol* 2007;**47**:412—17.
39. **Wong VW,** Wong GL, Yu J, *et al*. Interaction of adipokines and hepatitis B virus on histological liver injury in the Chinese. *Am J Gastroenterol* 2010;**105**:132—8.
40. **Wong VW,** Chan SL, Mo F, *et al*. Clinical scoring system to predict hepatocellular carcinoma in chronic hepatitis B carriers. *J Clin Oncol* 2010;**28**:1660—5.
41. **Bugianesi E,** Manzini P, D'Antico S, *et al*. Relative contribution of iron burden, HFE mutations, and insulin resistance to fibrosis in nonalcoholic fatty liver. *Hepatology* 2004;**39**:179—87.
42. **Nelson JE,** Wilson L, Brunt EM, *et al*. Relationship between the pattern of hepatic iron deposition and histological severity in nonalcoholic fatty liver disease. *Hepatology* 2011;**53**:448—57.
43. **Prati D,** Taioli E, Zanella A, *et al*. Updated definitions of healthy ranges for serum alanine aminotransferase levels. *Ann Intern Med* 2002;**137**:1—10.
44. **Kim HC,** Nam CM, Jee SH, *et al*. Normal serum aminotransferase concentration and risk of mortality from liver diseases: prospective cohort study. *BMJ* 2004;**328**:983.
45. **Mofrad P,** Contos MJ, Haque M, *et al*. Clinical and histologic spectrum of nonalcoholic fatty liver disease associated with normal ALT values. *Hepatology* 2003;**37**:1286—92.
46. **Wong VW,** Wong GL, Tsang SW, *et al*. Metabolic and histological features of non-alcoholic fatty liver disease patients with different serum alanine aminotransferase levels. *Aliment Pharmacol Ther* 2009;**29**:387—96.
47. **Hui AY,** Wong VW, Chan HL, *et al*. Histological progression of non-alcoholic fatty liver disease in Chinese patients. *Aliment Pharmacol Ther* 2005;**21**:407—13.
48. **Dunn W,** Xu R, Schwimmer JB. Modest wine drinking and decreased prevalence of suspected nonalcoholic fatty liver disease. *Hepatology* 2008;**47**:1947—54.
49. **Kechagias S,** Ernersson A, Dahlqvist O, *et al*. Fast-food-based hyper-alimentation can induce rapid and profound elevation of serum alanine aminotransferase in healthy subjects. *Gut* 2008;**57**:649—54.
50. **Younossi ZM,** Stepanova M, Rafiq N, *et al*. Pathologic criteria for nonalcoholic steatohepatitis: Interprotocol agreement and ability to predict liver-related mortality. *Hepatology* 2011;**53**:1874—82.

Downloaded from http://gut.bmj.com/ on June 14, 2015 - Published by group.bmj.com

GUT

Prevalence of non-alcoholic fatty liver disease and advanced fibrosis in Hong Kong Chinese: a population study using proton-magnetic resonance spectroscopy and transient elastography

Vincent Wai-Sun Wong, Winnie Chiu-Wing Chu, Grace Lai-Hung Wong, Ruth Suk-Mei Chan, Angel Mei-Ling Chim, Arlinking Ong, David Ka-Wai Yeung, Karen Kar-Lum Yiu, Shirley Ho-Ting Chu, Jean Woo, Francis Ka-Leung Chan and Henry Lik-Yuen Chan

Gut 2012 61: 409-415 originally published online August 16, 2011
doi: 10.1136/gutjnl-2011-300342

Updated information and services can be found at:
http://gut.bmj.com/content/61/3/409

These include:

References

This article cites 50 articles, 9 of which you can access for free at:
http://gut.bmj.com/content/61/3/409#BIBL

Email alerting service

Receive free email alerts when new articles cite this article. Sign up in the box at the top right corner of the online article.

Topic Collections

Articles on similar topics can be found in the following collections

Nonalcoholic steatosis (117)
Cirrhosis (320)

Notes

To request permissions go to:
http://group.bmj.com/group/rights-licensing/permissions

To order reprints go to:
http://journals.bmj.com/cgi/reprintform

To subscribe to BMJ go to:
http://group.bmj.com/subscribe/
