# Supplementary material for: Diet-Quality Scores and Prevalence of Nonalcoholic Fatty Liver Disease: A Population Study Using Proton-Magnetic Resonance Spectroscopy
Source: PLoS One. 2015 Sep 29;10(9):e0139310. doi: 10.1371/journal.pone.0139310 (PMC4587971; doi:10.1371/journal.pone.0139310)
Supplement: S2 File — (DOC) [file pone.0139310.s002.doc]

##

**The Chinese University of Hong Kong**

**Clinical Study Protocol**

**Prevalence of nonalcoholic fatty liver disease and advanced liver fibrosis in Hong Kong – A cross-sectional population study using *in vivo* proton magnetic resonance spectroscopy and transient elastography (Fibroscan)**

**Principal investigator: Vincent W.S. Wong**

**Associate Professor**

**Department of Medicine and Therapeutics**

**The Chinese University of Hong Kong**

**Co-investigators: Winnie C.W. Chu**

**Professor**

**Department of Diagnostic Radiology and Organ Imaging**

**The Chinese University of Hong Kong**

**David K.W. Yeung**

**Physicist**

**Department of Clinical Oncology**

**Medical Physics Division**

**Prince of Wales Hospital**

**Henry L.Y. Chan**

**Professor**

**Department of Medicine and Therapeutics**

**The Chinese University of Hong Kong**

**Francis K.L. Chan**

**Professor**

**Department of Medicine and Therapeutics**

**The Chinese University of Hong Kong**

**1. BACKGROUND**

Nonalcoholic fatty liver disease (NAFLD) is the most common chronic liver disease in affluent countries.^1^ The estimated prevalence in Asia is between 15% to 30%.^2^ It may progress to cirrhosis and liver cancer. In Chinese NAFLD patients, advanced fibrosis and significant necroinflammation occur in up to 10% to 20% of cases.^3, 4^ NAFLD is closely associated with metabolic syndrome and central obesity.^5^

However, the understanding of the epidemiology of NAFLD is incomplete because previous studies have a number of limitations. Most population studies used ultrasound scan for the diagnosis of fatty liver.^6^ Ultrasound scan is operator dependent, and has limited sensitivity and specificity for the diagnosis of fatty liver.^7^ Besides, ultrasound scan cannot be used to assess necroinflammation and fibrosis.^7^ On the other hand, studies using liver biopsies only included a biased group of patients with abnormal liver function tests or high risk features. It is not feasible or ethical to perform liver biopsies in the general population. Therefore, the exact impact of NAFLD at the population level is currently unknown.

With advances in technology, it is possible to fill in the gap of knowledge. Firstly, the proton magnetic resonance spectroscopy (^1^H-MRS) is a sensitive and quantitative noninvasive technique to measure hepatic triglyceride content.^8^ This technique has been successfully used in population screening.^9^ Secondly, transient elastography by Fibroscan is an accurate noninvasive technique to measure hepatic fibrosis in a variety of liver diseases.^10-13^ The combination of these two techniques allow us to assess the prevalence of NAFLD and NAFLD complicated with advanced fibrosis at the same time.

**2. AIM**

The aim of this study is to assess the prevalence of NAFLD and advanced fibrosis in Hong Kong.

**3. STUDY DESIGN OVERVIEW**

This is a cross-sectional study. Subjects from the general population will be invited to have comprehensive anthropometric and biochemical assessment. ^1^H-MRS and transient elastography will be performed to assess the degree of hepatic triglyceride content and hepatic fibrosis, respectively.

**4. SUBJECT SELECTION**

**4.1 Source of patients**

In 2003, our center conducted a population study on the seroprevalence for severe acute respiratory syndrome coronavirus.^14^ Residents of Hong Kong were randomly selected by computer. Twelve thousand subjects participated in the project and consented to be contacted for future studies. In this study, we plan to randomly select subjects from this cohort and invite them for screening.

**4.2 Inclusion criteria**

Patients would be eligible if they fulfill all of the following criteria:

- Age 18 to 70 years
- Informed written consent obtained

**4.3 Exclusion criteria**

Patients would be excluded from the current study if they have any of the following:

- Decompensated liver disease, defined as bilirubin >50 µmol/l, albumin <35 g/l, platelet count <150 × 10^9^/l, INR >1.3, or the presence of ascites or varices
- Presence of medical implants that are contra-indicated for magnetic resonance imaging

**5. STUDY PROCEDURES**

**5.1 Baseline clinic visit**

A baseline visit will be performed to determine suitability for the subject’s inclusion in the study. Clinical assessment will be performed after verifying the conformance with entry inclusion and exclusion criteria, and obtaining informed written consent. Afterwards, the following procedure will be performed:

- Record medical history including medication history and past medical history
- Physical examination including measurement of subject’s height, weight, waist circumference, hip circumference, blood pressure and pulse rate
- Questionnaire on smoking, alcohol, caffeine consumption and diet
- Clinical laboratory tests including liver function, renal function, aspartate aminotransferase, fasting glucose, lipids, LDL-cholesterol, HDL-cholesterol
- 10 ml clotted blood would be saved for special assays including adiponectin and fasting insulin level
- Transient elastography by Fibroscan
- Book ^1^H-MRS

**5.2 Transient elastography**

Transient elastography is performed during the baseline clinic visit according to the instructions and training provided by the manufacturer. Ten successful acquisitions will be performed on each patient. The success rate is calculated as the ratio of the number of successful acquisitions over the total number of acquisitions. The median value is kept as representative of the liver elastic modulus. The liver stiffness measurements are considered reliable only if 10 successful acquisitions are obtained and success rate is above 60%. The liver stiffness is expressed in kiloPascal (kPa). A higher kPa reflects a stiffer liver and more severe liver fibrosis. Operators in this study should have performed 50 procedures in the past, as operators with this kind of experience were found to have the best success rate and reproducibility in LSM.^15^

**5.3 Magnetic resonance examination**

**5.3.1 *In Vivo* ^1^H-MRS**

Fat measurement in the liver will be performed on a whole-body 3.0T scanner using a single voxel point-resolved spectroscopy sequence (PRESS) with an echo-time (TE) of 40 ms and repetition time (TR) of 5000 ms. A survey scan will first be performed in the abdominal region to help positioning a volume measuring 20 (AP) x 15 (RL) x 40 (FH) mm within the liver. The scanner’s built-in body coil will be used for both signal transmission and reception. No-water-suppressed spectra will be acquired using 32 signal averages and data will be exported for off-line spectral analysis. Water (4.65 ppm) and lipid (1.3 ppm) peak amplitudes will be measured to determine vertebral marrow fat content, which is defined as the relative fat signal amplitude in terms of a percentage of total signal amplitude (water and fat) and calculated according to the following equation: fat content =[*I*_fat_/(*I*_fat_+*I*_water_)]x100, where *I*_fat_ and *I*_water_ are the peak amplitudes of fat and water, respectively. No correction for relaxation losses will be applied because of the relatively long TR and short TE employed.

**5.3.2 MR Imaging of Visceral Adioposity**

For the measurement of abdominal fat, we will use a whole-body 3.0T scanner to obtain high-resolution T1-weighted MR images (axial scan, 10 mm thick, 30 slices, no gap) to show the distribution of visceral fat in the abdominal cavity of each patient. Patients will be instructed to hold their breath for 10-15 seconds when image acquisition is in progress to minimize motion artifact seen on MR images. Fatty tissues appear as hyper-intense on T1-weighted images and an image segmentation technique will be used to measure the cross-sectional area of fatty tissues in each axial image. Fatty tissue volume will then be measured as the sum of all fatty tissue areas multiplied by slice thickness.

**5.4 Follow-up clinic visit**

A follow-up clinic visit will be arranged 4 to 6 weeks after the ^1^H-MRS. The laboratory results will be discussed with the patients. People with simple steatosis run a benign clinical course, and those with significant fibrosis may progress to cirrhosis and have increased mortality rate. Therefore, we will follow up patients with NAFLD and abnormal transient elastography at our research clinic. For patients with NAFLD but no significant fibrosis, we will give counseling at the follow-up visit and discharge them back to the community. Similarly, if abnormal blood tests (e.g. abnormal renal, liver function or lipid profile) are detected, we will refer the subjects to the regional hospitals or general out-patient clinics as appropriate. Before the subjects can be seen at those clinics, we will assume necessary follow-up action at our research clinic. The arrangement will not affect the waiting list of the current clinics of the Hospital Authority.

**6. ENDPOINT ASSESSMENT**

**6.1 Primary endpoint**

The primary endpoint is the prevalence of NAFLD as determined by the ^1^H-MRS.

**6.2 Secondary endpoints**

Secondary endpoints include the prevalence of advanced hepatic fibrosis as determined by transient elastography and the prevalence of advanced hepatic fibrosis among NAFLD patients.

**7. SAFETY**

**7.1 Blood taking**

Blood taking is a safe procedure. Occasionally, bruises may occur at the venepuncture site.

**7.2 Transient elastography**

Transient elastography is a non-invasive procedure. It is pain free and has no major complications.

**7.3 Magnetic resonance imaging**

Magnetic resonance examination is a non-invasive procedure that does not involve ionizing radiation. The examination is painless and this study will not require patients to be injected with contrast enhancing material. Rarely, patients may develop panic attacks in the magnetic resonance imaging machine.

**8. DATA ANALYSIS AND STATISTICAL METHODS**

**8.1 Sample size determination**

According to the census in 2006, the Hong Kong population with age 15 years or above is 5,924,671.^16^ A sample size of 1067 will determine the prevalence of NAFLD in Hong Kong at a 3% confidence interval and 95% confidence level.[Sample size calculator, <http://www.surveysystem.com/sscalc.htm>]

**8.2 Statistical analysis**

Unpaired t test, chi-square test and Fisher exact test will be used to compare subjects with and without NAFLD, and with and without advanced fibrosis, where appropriate. Logistic regression analysis (univariate and multivariate) will be performed to identify factors associated with NAFLD and advanced fibrosis.

**9. References**

1. Farrell GC, Chitturi S, Lau GK, Sollano JD. Guidelines for the assessment and management of non-alcoholic fatty liver disease in the Asia-Pacific region: executive summary. J Gastroenterol Hepatol 2007;22:775-7.

1. Amarapurkar DN, Hashimoto E, Lesmana LA, Sollano JD, Chen PJ, Goh KL. How common is non-alcoholic fatty liver disease in the Asia-Pacific region and are there local differences? J Gastroenterol Hepatol 2007;22:788-93.
2. Wong VW, Chan HL, Hui AY, et al. Clinical and histological features of non-alcoholic fatty liver disease in Hong Kong Chinese. Aliment Phar Ther. 2004;20:45-9.
3. Wong VW, Hui AY, Tsang SW, et al. Prevalence of undiagnosed diabetes and postchallenge hyperglycemia in Chinese patients with non-alcoholic fatty liver disease. Aliment Phar Ther 2006;24:1215-22.
4. Wong VW, Hui AY, Tsang SW, et al. Metabolic and adipokine profile of Chinese patients with nonalcoholic fatty liver disease. Clin Gastroenterol Hepatol 2006;4:1154-61.
5. Fan JG, Zhu J, Li XJ, et al. Prevalence of and risk factors for fatty liver in a general population of Shanghai, China. J Hepatol 2005;43:508-14.
6. Saadeh S, Younossi ZM, Remer EM, et al. The utility of radiological imaging in nonalcoholic fatty liver disease. Gastroenterology 2002;123:745-50.
7. Szczepaniak LS, Nurenberg P, Leonard D, et al. Magnetic resonance spectroscopy to measure hepatic triglyceride content: prevalence of hepatic steatosis in the general population. Am J Physiol Endocrinol Metab 2005;288:E462-8.
8. Browning JD, Szczepaniak LS, Dobbins R, et al. Prevalence of hepatic steatosis in an urban population in the United States: impact of ethnicity. Hepatology 2004;40:1387-95.
9. Ganne-Carrie N, Ziol M, de Ledinghen V, et al. Accuracy of liver stiffness measurement for the diagnosis of cirrhosis in patients with chronic liver diseases. Hepatology 2006;44:1511-7.
10. Talwalkar JA, Kurtz DM, Schoenleber SJ, et al. Ultrasound-based transient elastography for the detection of hepatic fibrosis: systematic review and meta-analysis. Clin Gastroenterol Hepatol 2007;5:1214-20.
11. Wong GL, Wong VW, Choi PC, et al. Assessment of fibrosis by transient elastography compared with liver biopsy and morphometry in chronic liver diseases. Clin Gastroenterol Hepatol 2008 (in press).
12. Wong VW, Wong GL, Chim AM, et al. Combination of the median and variability of liver stiffness measurements by transient elastography improves the accuracy of diagnosing advanced fibrosis in nonalcoholic fatty liver disease. J Hepatol 2008 (abstract in press).
13. Leung DT, van Maren WW, Chan FK, et al. Extremely low exposure of a community to severe acute respiratory syndrome coronavirus: false seropositivity due to use of bacterially derived antigens. J Virol 2006;80:8920-8.
14. Fraquelli M, Rigamonti C, Casazza G, et al. Reproducibility of transient elastography in the evaluation of liver fibrosis in patients with chronic liver disease. Gut 2007;56:968–973.
15. Census and Statistics Department. Population by age group, 1996, 2001 and 2006. <http://www.censtatd.gov.hk/hong_kong_statistics/statistical_tables/index.jsp?charsetID=1&subjectID=1&tableID=137> (accessed on 30 March 2008).
